# Torque Teno Virus load in lung cancer patients correlates with age but not with tumor stage

**DOI:** 10.1371/journal.pone.0252304

**Published:** 2021-06-02

**Authors:** Dirk Stefani, Balazs Hegedues, Stephane Collaud, Mohamed Zaatar, Till Ploenes, Daniel Valdivia, Carina Elsner, Barbara Bleekmann, Marek Widera, Ulf Dittmer, Clemens Aigner

**Affiliations:** 1 Department of Thoracic Surgery, Ruhrlandklinik, University of Duisburg-Essen, Essen, Germany; 2 Institute of Virology, University Hospital Essen, University of Duisburg-Essen, Essen, Germany; 3 Institute of Medical Virology, University Hospital Frankfurt am Main, Goethe University, Frankfurt am Main, Germany; Gustave Roussy, FRANCE

## Abstract

**Background:**

Torque teno virus (TTV) is a ubiquitous non-pathogenic virus, which is suppressed in immunological healthy individuals but replicates in immune compromised patients. Thus, TTV load is a suitable biomarker for monitoring the immunosuppression also in lung transplant recipients. Since little is known about the changes of TTV load in lung cancer patients, we analyzed TTV plasma DNA levels in lung cancer patients and its perioperative changes after lung cancer surgery.

**Material and methods:**

Patients with lung cancer and non-malignant nodules as control group were included prospectively. TTV DNA levels were measured by quantiative PCR using DNA isolated from patients plasma and correlated with routine circulating biomarkers and clinicopathological variables.

**Results:**

47 patients (early stage lung cancer n = 30, stage IV lung cancer n = 10, non-malignant nodules n = 7) were included. TTV DNA levels were not detected in seven patients (15%). There was no significant difference between the stage IV cases and the preoperative TTV plasma DNA levels in patients with early stage lung cancer or non-malignant nodules (p = 0.627). While gender, tumor stage and tumor histology showed no correlation with TTV load patients below 65 years of age had a significantly lower TTV load then older patients (p = 0.022). Regarding routine blood based biomarkers, LDH activity was significantly higher in patients with stage IV lung cancer (p = 0.043), however, TTV load showed no correlation with LDH activity, albumin, hemoglobin, CRP or WBC. Comparing the preoperative, postoperative and discharge day TTV load, no unequivocal pattern in the kinetics were.

**Conclusion:**

Our study suggest that lung cancer has no stage dependent impact on TTV plasma DNA levels and confirms that elderly patients have a significantly higher TTV load. Furthermore, we found no uniform perioperative changes during early stage lung cancer resection on plasma TTV DNA levels.

## Introduction

The ubiquitous Torque teno virus (TTV) has a high prevalence of about 90% in the population worldwide [1–3]. It was initially detected in 1997 in a patient with post transfusion hepatitis in Japan [4]. TTV is a single stranded circular Anellovirus from the Circoviridae family of about 3.8 kilobases [5]. It leads to a low viremia in immune competent hosts [6]. TTV viral load increases during the first months of healthy infants development [7].

While TTV infection had been suggested to be associated epidemiologically with many diseases like respiratory [8], hepatic [9] or hematological disorders [10] and cancer [11], a direct causal link between TTV infection and specific diseases is lacking. Therefore, the overall consensus now is that TTV is nonpathogenic [6]. However, TTV DNA load was demonstrated as a suitable surrogate marker for immune competence [12–17]. TTV DNA plasma loads were found to be elevated in patients under immunosuppression after lung transplantation and other solid organ transplantations [16, 18]. The extent of TTV viremia correlates with the state of immunosuppression of the transplant recipient [19] and works as a predictor for the development of rejection and infection in lung transplantation [15]. TTV plasma DNA levels can therefore be used as a marker for monitoring the extent of immunosuppression in lung [20], kidney [21] and liver transplant recipients [22].

The association of TTV DNA levels with carcinogenesis and clinical course of lung cancer is poorly understood. TTV DNA has been found in a variety of neoplastic tissues including lung cancer [11]. Furthermore, TTV load was significantly higher in the peripheral blood mononuclear cells from cancer patients with various malignancies than in the PBMC from healthy blood donors [23]. Previously it has been demonstrated that patients suffering from both idiopathic pulmonary fibrosis (IPF) and lung cancer have higher TTV DNA levels than lung cancer only or IPF only patients. Of note, TTV loads did not match with tumor stages among these patients with lung cancer complicated with IPF [8]. Among platinum based chemotherapy treated advanced stage lung cancer patients the TTV DNA levels decreased after therapy in the partial response/stable disease subgroup and increased in the progressive disease subcohort which led to the hypothesis that high TTV loads may correlate with higher tumor load and thus with tumor stage [24].

In chronically infected individuals, TTV viremia fluctuates very little over periods of weeks or months indicative of the existence of a quasi-steady-state virus-host equilibrium resulting from balanced virus production and elemination. However, the dynamic nature of TTV viremia is highlighted by the observation that over 90% of the TTV virions found in the plasma of chronically infected subjects turns over daily [25]. Furthermore, significant changes of TTV load can be detected as early as three days after alpha-interferon treatment in Hepatitis C patients [25].

Accordingly, we analyzed the TTV plasma DNA load in early and advanced stage lung cancer patients as well as monitored its perioperative changes after curative intent resection.

## Materials and methods

### Patients

47 patients were included prospectively in the study from January 2019 to January 2020. Current immunosuppressive medication and chemotherapy within the previous 12 months were exclusion criteria. 30 patients with early stage lung cancer underwent curative intent surgery (23 (bi-) lobectomy, 4 sleeve-lobectomy, 3 segmentectomy). Resected lung tumors were pathologically staged (pT and pN) by using the UICC 8^th^ edition of lung cancer TNM staging. 10 patients had stage IV lung cancer and tumor size (cT) and lymph node involvement (cN) was assessed by radiological staging. In the non-malignant group diagnoses included hamartochondroma (n = 2), organizing pneumonia (n = 3) and necrotizing granulomatosis (n = 2). Pathological tumor stage, histology, procedure, operation time, blood loss, major and relevant pre-existing conditions or events and postoperative complication of the patients under investigation were registered. Routine blood parameters including amounts of white blood cell counts (WBC), concentrations of albumin, hemoglobin, lactate dehydrogenase (LDH) and C-reactive protein (CRP) were also measured preoperatively. Plasma was collected prior to the operation, one day after surgery and at discharge (4 to 9 days). Plasma samples were collected from stage IV patients once during their hospital stay for staging purposes but prior to any procedure. All plasma samples were collected in collaboration with the Westgerman Biobank Essen (WBE). Every patient provided written informed consent. The study was approved by the Ethics Committee of the University Duisburg-Essen (#18–8539) and was conducted in compliance with the Declaration of Helsinki.

### Quantification of TTV DNA

TTV DNA was isolated from 200 μl of patient plasma using the QIAamp DNA Blood Mini Kit (Qiagen, Hilden, Germany; #51106) following the manufacturer’s instruction. DNA was subjected to real-time PCR analysis as described [16, 17, 26] using the Rotor-Gene Probe PCR Kit (Qiagen, #204374). TTV specific primers and a 5’FAM / 3’TAMRA labeled probe [26] were purchased from metabion GmbH (Planegg/Steinkirchen, Germany). As a positive control for DNA quality we used human GAPDH specific primers and a 5’YAK/3’BBQ labeled probe purchased from TIB MOLBIOL (Berlin, Germany). The primer sequences were as follows according to PrimerBank ID 378404907c1: forward 5’GGAGCGAGATCCCTCCAAAAT, reverse 5’ GGCTGTTGTCATACTTCTCATGG. The probe has the following sequence: 5’ YAK-AGTGGGGCGATGCTGGCGCTGAG-BBQ. For absolute quantification we used GAPDH cDNA kindly provided by Dr. Kathrin Sutter (University Hospital Essen, Essen, Germany) to generate a standard curve. The cycling conditions on a Rotor-Gene-Q-Instrument (Qiagen) were as follows: initial denaturation was 10 min at 95°C followed by 45 cycles of denaturation for 15 seconds and extension for 60 seconds at 58°C. As described previously, TTV genotype 1a DNA (AB017610.1) cloned into a pCR2.1 vector was used to generate a DNA standard curve [16, 17, 26]. The limit of quantification (LOQ) was 50 copies per ml. Samples with copy numbers below the quantification limit were included in calculations with a value of LOQ/2 (i.e. 25 copies per ml) [27].

### Statistical analysis

For continuous variables the normality of distribution was tested by Shapiro-Wilk test. For comparing two groups Mann-Whitney test was used. One-way analysis of variance (ANOVA) was performed for normally distributed paramaters (age, albumin, hemoglobin) and Kruskal-Wallis test (TTV DNA levels, LDH) with Dunn’s multiple comparison test were used as non-parametric analysis. Correlation of TTV DNA levels with amounts of WBC, albumin, hemoglobin and LDH was analyzed by Spearman test. Contingency analysis of the three patient subcohort for categorical variables (gender, tumor stage, CRP) was compared with chi-square test. P-values of p <0.05 were considered as significant. Statistical analysis was conducted using GraphPad Prism 5.0 software.

## Results

The major clinicopathological characteristics and biomarker values for the early stage metastatic lung cancer patients as well as for the non malignant lung nodule patients are presented in Table 1.

**Table 1:** Clinicopathological characteristics of the patient subgroups.

		Early stage lung cancer (n = 30)	Stage IV lung cancer (n = 10)	Non-malignant (n = 7)
Gender	Male	20	7	2
	Female	10	3	5
Age	Mean±SD	67.4±8.3	61.7±8.1	69.3±10.3
Operation	VATS	15	n.a.	6*
	Thoracotomy	15		0
Histology	Adenocarcinoma	12	8	n.a.
	Squamous cell	13	2	
	Adenosquamous	3		
	Neuroendocrine	2		
Stage (UICC 8th Edition)	IA	15		n.a.
	IB	3		
	IIA	4		
	IIB	4		
	IIIA	4		
	IVA		5	
	IVB		5	
pT descriptor	1	16	0	n.a.
	2	9	3	
	3	2	2	
	4	3	5	
pN descriptor	0	26	1	n.a.
	1	4	0	
	2	0	7	
	3	0	2	
CRP	<1 mg/dl	19	4	6
	>1 mg/dl	11	6	1
albumin	Mean±SD [g/dL]	4.4±0.3	4.15±0.5	4.5±0.2
hemoglobin	Mean±SD [g/dL]	14.1±1.6	12.5±1.6	13.3±1.5
WBC	Mean±SD [10^9^/L]	8.3±2.4	8.6±3	8.1±3.2
LDH	Mean±SD [U/L]	222±49	286±77	231±72

*one patient had no operation

VATS–Video Assisted Thoracic Surgery; CRP–C-reactive protein; WBC–white blood cells; LDH–lactate dehydrogenase; SD–standard deviation; n.a.–not applicable

The early stage subgroup (stage I-III) was representative for a curative intent resection cohort in terms of histology, access and stage distribution. There was no statistically significant difference between the three subgroups regarding gender (p = 0.146) and age (p = 0.134) or white blood cell counts (p = 0.92*)*. High CRP values (>1 mg/dl) tended to be more frequent in stage IV lung cancer patients (p = 0.066). LDH was significantly increased in stage IV lung cancer cases (p = 0.043, Table 1).

**Fig 1:**
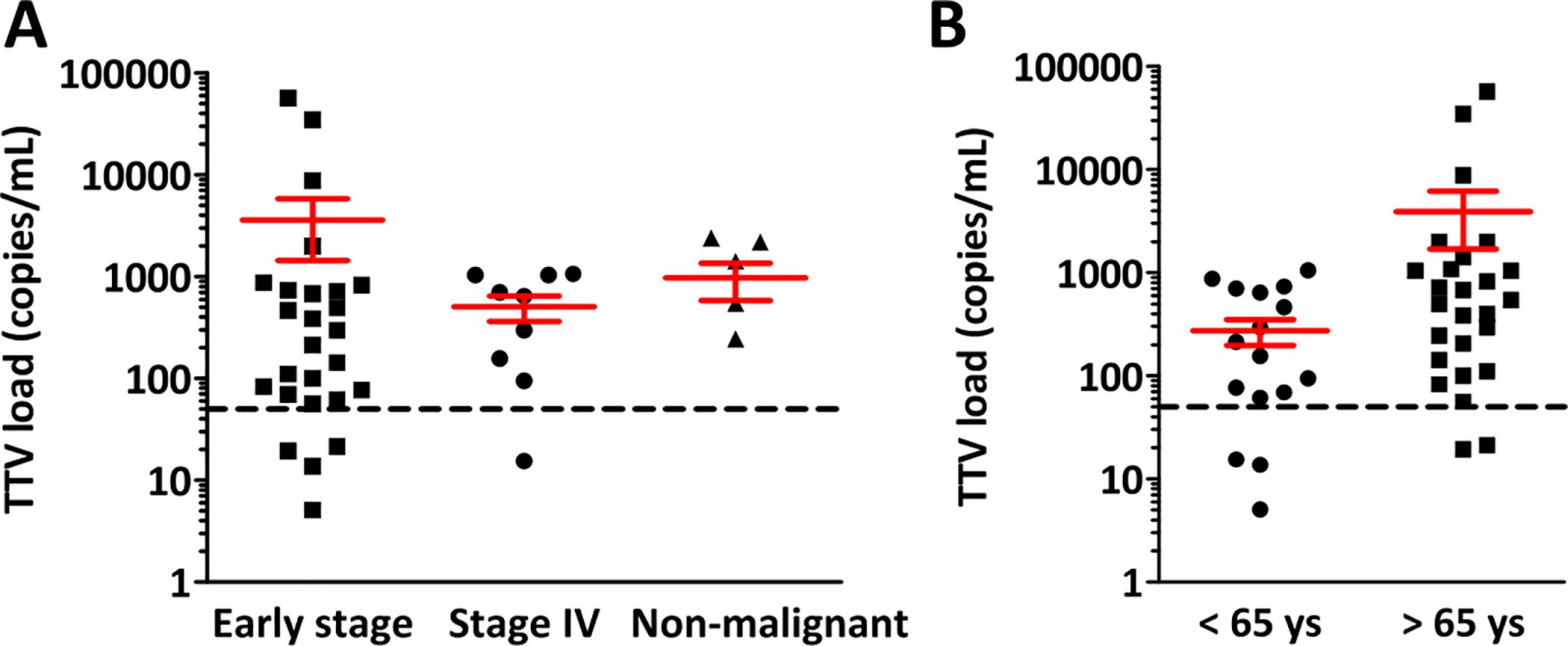
Baseline TTV DNA levels. *(A)* There was no significant difference in the TTV copy numbers in the blood of arly stage and stage IV lung cancer patients and patients without malignant lung tumors. *(B)* Patients older than 65 years of age had significantly lower TTV DNA levels in the blood (p = 0.022).

TTV DNA was not detectable in the blood of 7 patients (15%) including 4 early stage, 1 stage IV and 2 non-malignant cases. In all samples tested, GAPDH was amplified as a control and the GAPDH levels were comparable throughout the samples. TTV viral load did not show significant differences between early stage and advanced stage cases or in patients without malignancy (p = 0.63, Fig 1A). There was no significant difference in the TTV viral load between male and female patients (p = 0.634). In contrast patients younger than 65 years of age had significantly lower TTV viral loads than older patients (p = 0.022, Fig 1B).

**Fig 2:**
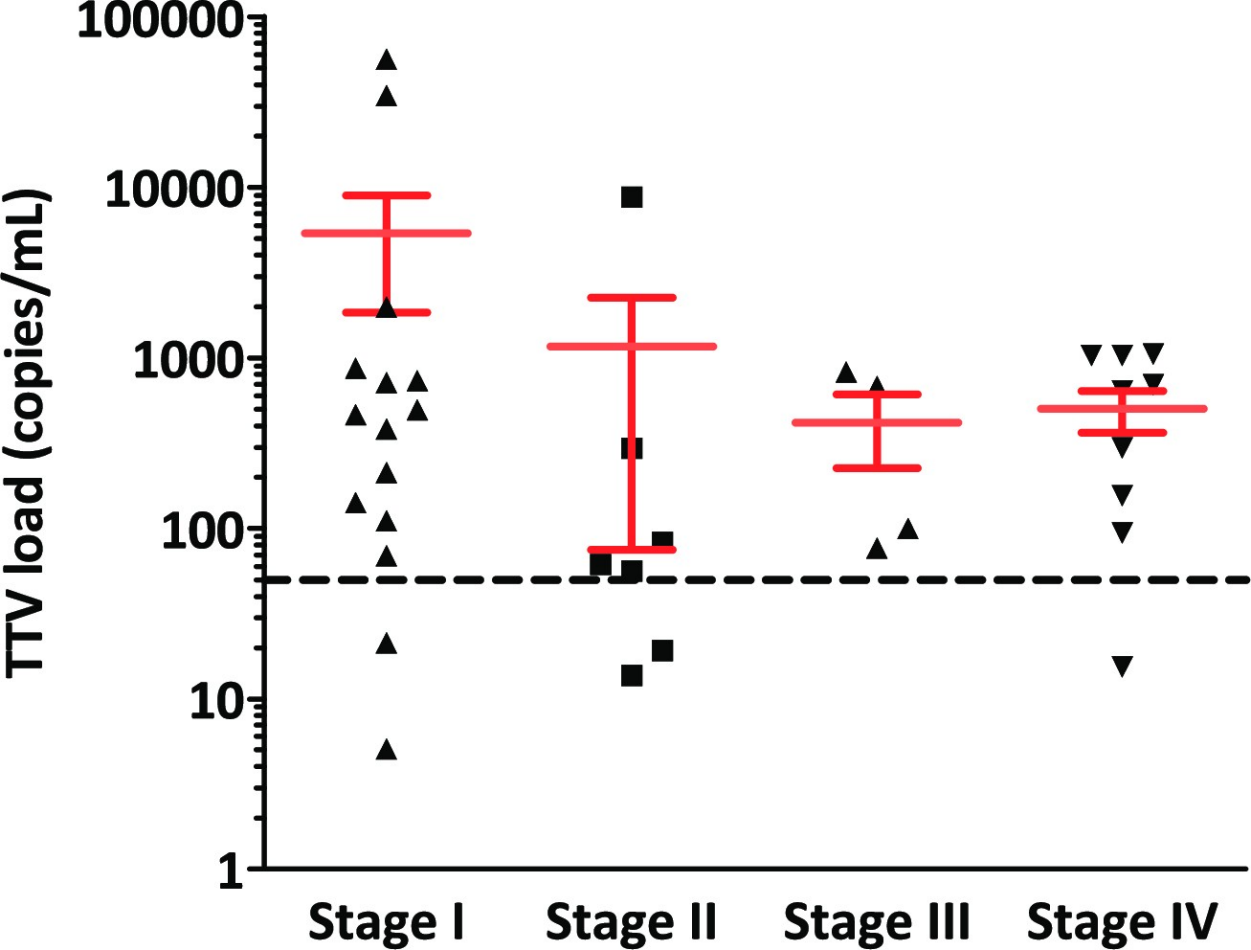
TTV DNA levels in the lung cancer patients grouped by TNM stage (UICC 8th edition). There was no significant difference in the 4 stage groups (p = 0.436).

TTV DNA viral load was also independent of histology (adenocarcinoma versus squamous cell carcinoma, p = 0.433). The pathological TNM tumor stage was defined by the UICC 8^th^ edition. The TTV viral load showed no significant differences in the four TNM stage group (Fig 2, p = 0.436).

**Fig 3:**
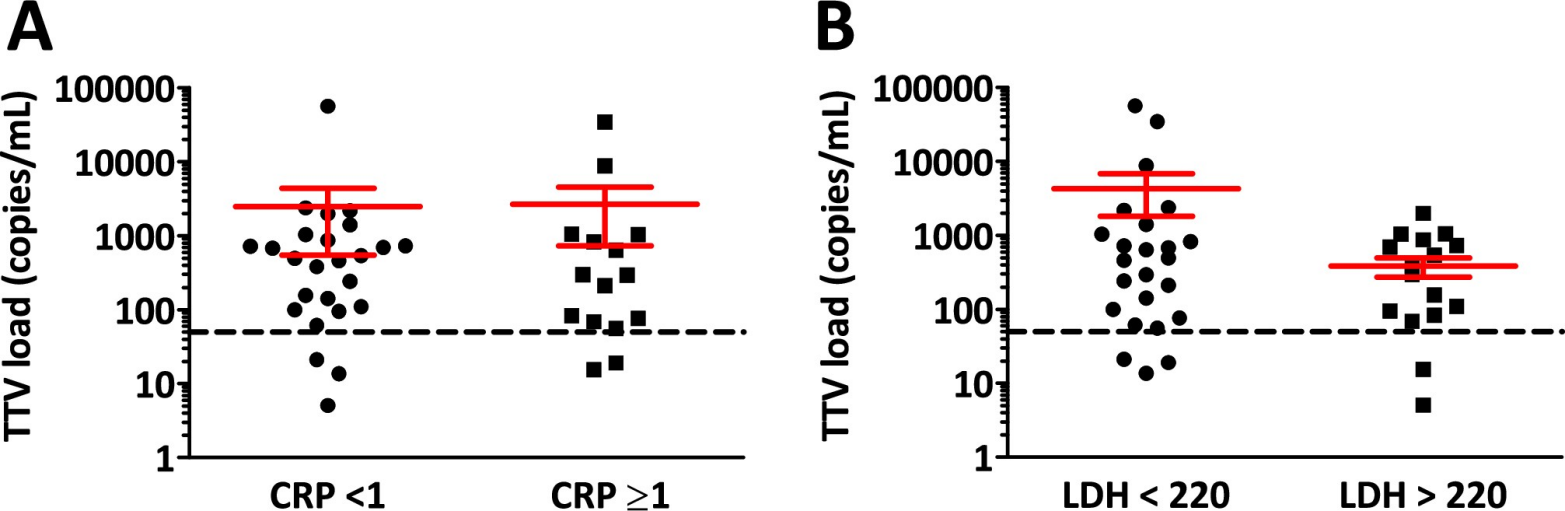
Baseline TTV DNA levels in patients with normal and increased CRP or LDH. (A, B) There was no significant difference between the two groups, (p = 0.547 and p = 0.155).

Furthermore, TTV load showed no correlation with albumin, hemoglobin, LDH and WBC values (p = 0.781, p = 0.295, p = 0.127 and p = 0.608, respectively). There was no significant difference in TTV copy numbers between CRP high (≥1) and low (<1) (p = 0.547) or LDH high (>220) and low (<220) patients (p = 0.155, Fig 3).

**Fig 4:**
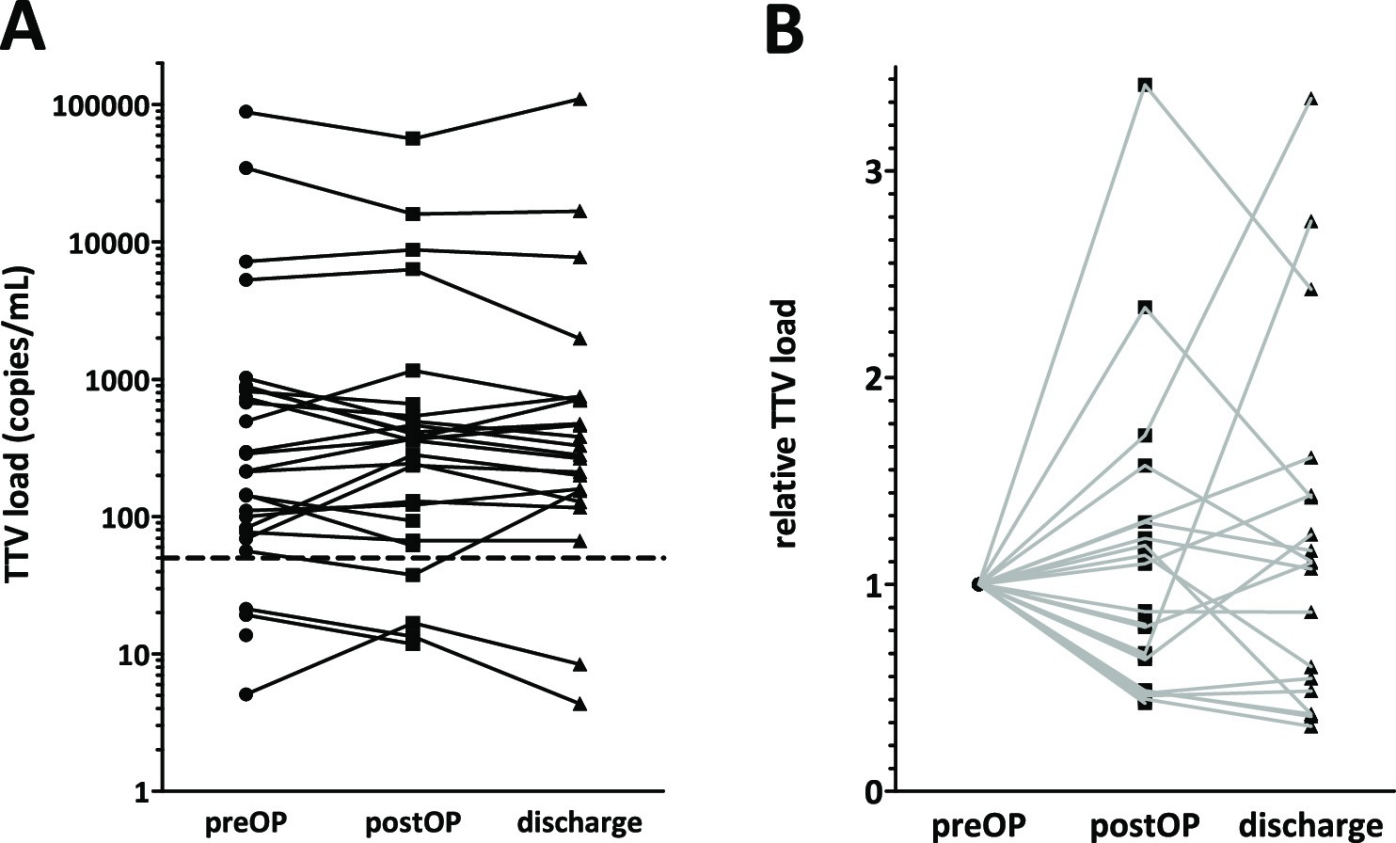
Perioperative changes in TTV DNA levels during resection of early stage lung tumors prior to the operation, on the first postoperative day and at discharge. (A) Absolut TTV DNA copy numbers showed minor changes in TTV plasma DNA levels. (B) Relative TTV loads normalized to the preoperative value shows no unequivocal direction of changes on postoperative day 1 or at discharge.

Next, we analyzed whether the resection has an impact on TTV DNA levels. Absolute and relative TTV loads in early lung cancer patients and its perioperative changes after lung cancer surgery are shown in Fig 4. Preoperative TTV plasma DNA load showed a very wide range in lung cancer patients. Postoperative changes of TTV plasma DNA levels remained well below an order of magnitude (Fig 4A). 4 patients had a more than 50% increase and 7 patients a slightly more than 50% decrease in relative TTV plasma DNA load at postoperative day 1 (Fig 4B).

At discharge 4 and 6 patients demonstrated 50% increase or decrease in relative TTV plasma DNA levels, respectively.

## Discussion

In the current study we measured the TTV plasma DNA levels in a lung cancer patient cohort and in a group of patients with non malignant lung nodules or other non malignant indication for lung operation. 15% of all patients had no detectable levels of TTV DNA in the DNA isolated from the patient’s plasma comparable to previous prevalence studies using blood donor collectives [3, 28].

In our patient population, older individuals had significantly higher TTV DNA levels. This finding is in line with previous investigations on smaller cohorts and with a large survey for markers of aging (MARK-AGE) [29–31]. Indeed, in the MARK-AGE study only age and gender showed a significant association with TTV loads but no blood based routine laboratory parameter did. We found no correlation between TTV load and gender, however, only 17 female patients (36%) participated in our study. Haloschan et al found lower TTV levels for women under the age of 30 when compared to men [31] but our patient cohort includes only patients older than 30 years of age. Confirming the findings of the MARK-AGE study [29], TTV DNA levels showed no significant association with the amount of hemoglobin, albumin, CRP or WBC in our cohort.

De Villiers et al found varying TTV positivity in tumor tissue samples from different types of cancer, however, the study did not compare different histologies within lung cancer [11]. Nevertheless, there is currently no data available whether lung cancer histology shows an association with TTV viral load in the patients plasma. Our case series consisted primarily from adenocarcinoma and squamous cell carcinoma, however, the lack of significant difference between the histologies should be carefully interpreted due to the relatively low case number.

Since TTV DNA load is a surrogate marker for immune competence in transplant patients [13, 15, 17, 18], we hypothesized that potential differences in immunocompetence based on the presence or disease stage of lung cancer might impact TTV DNA load in the patient’s blood. However, we found no difference between the subcohorts with or without lung cancer. In line with our findings, Bando et al reported no differences between lung cancer patients and indiopathic pulmonary fibrosis (IPF) patients [8]. Interestingly, the aforementioned report found significantly higher TTV DNA titers in lung cancer patients with IPF. In our study disease stage showed no association with circulating TTV load confirming the previous report [8]. In this earlier study the majority of patients were in stage IV, in contrast to our study where the majority was early stage lung cancer. Nevertheless, both study supports the notion that circulating TTV DNA levels seem to be disease stage independent in lung cancer.

Regarding the association of TTV loads with the outcome of lung cancer therapy Sawata et al reported that TTV DNA levels decreased after platinum based chemotherapy treatment of advanced stage patients with partial response or stable disease. In contrast, TTV loads increased after chemotherapy in patients with progressive disease [24]. Due to the short follow-up time in our series, we can not investigate whether TTV DNA baseline values or its changes after surgery might correlate with outcome after curative intent lung resection. Advanced stage patients are the younger patients than patients in the early stage and non malignant subgroup in our study. Therefore the TTV levels are in general lower because of the lower age, which could counteract and disguise a possible high TTV load in these patients.

The kinetics of TTV DNA during tumor progression is yet to be studied. Regarding the dynamics of TTV load, Maggi et al. calculated a more than 90 percent daily turnover for circulating TTV DNA and found significant decrease of plasma TTV levels already after three days of interferon treatment in Hepatitis C patients [25]. There are a couple of publications investigating changes in TTV loads after lung transplantation [15, 18]. In the majority of lung transplant recipients within two weeks after transplantation a marked increase can be observed due to immunosuppression. In cancer and specifically in lung cancer, the comparison of TTV DNA copy numbers before and after chemotherapy in a small advanced stage cohort demonstrated that depending on response to therapy the extent of TTV viremia can change in either direction [24]. In our early stage lung cancer patients there was no clear tendency for a decrease or increase of plasma TTV DNA levels on postoperative day 1 or at discharge (4 to 9 postoperative days). A previous study indicated that TTV DNA load in the peripheral blood mononuclear cells of cancer patients (not specifically lung cancer patients) is significantly higher when compared to PBMC from healthy donors [23]. Altogether, it remains an open question whether the tumor itself is a major site of viral replication. Long-term follow-up studies are warranted in order to investigate whether tumor resection or potentially tumor recurrence or progression has an impact on TTV loads.

## Conclusion

Torque teno virus load in lung cancer patients does not assoicate with tumor stage in this study but is increased in elderly patients. Moreover, we found no correlation of TTV DNA levels with routine laboratory parameters. Finally, we identified perioperative changes in TTV viral load in patients undergoing curative intent lung cancer resection, however, the perioperative kinetics showed no unequivocal pattern.

## Supporting information

S1 File (XLSX)
